# Dynamic Changes in Egg Quality, Heritability and Correlation of These Traits and Yolk Nutrient throughout the Entire Laying Cycle

**DOI:** 10.3390/foods12244472

**Published:** 2023-12-14

**Authors:** Junnan Zhang, Xiang Gao, Weijie Zheng, Pengpeng Wang, Zhongyi Duan, Guiyun Xu

**Affiliations:** 1 State Key Laboratory of Animal Biotech Breeding and Frontier Science Center for Molecular Design Breeding, National Engineering Laboratory for Animal Breeding and Key Laboratory of Animal Genetics, Breeding and Reproduction, Ministry of Agriculture and Rural Affairs, Department of Animal Genetics and Breeding, College of Animal Science and Technology, China Agricultural University, Beijing 100193, China; cauzhangjn@163.com (J.Z.); weijiezheng426@163.com (W.Z.); 2 Key Laboratory of Animal Epidemiology of the Ministry of Agriculture and Rural Affairs, College of Veterinary Medicine, China Agricultural University, Beijing 100193, China; stu2088@163.com; 3 Police-Dog Technology Department, Criminal Investigation Police University of China, Shenyang 110034, China; 18754881870@163.com; 4 National Animal Husbandry Service, Ministry of Agriculture and Rural Affairs, Beijing 100125, China; dzy806@163.com

**Keywords:** native breed, aging, egg quality, genetic parameters, nutrient content

## Abstract

Egg quality and nutritional value are becoming increasingly important to consumers, offering a new direction for the development of high-quality eggs. In this study, we conducted a comprehensive analysis of egg quality and nutrient profiles in native breeds at different ages, integrating pedigree data. Our results reveal dynamic changes in egg qualities, stronger associations among eggshell-related traits, and the effect of onset production and body weight on egg qualities. The heritability of different traits was estimated, ranging from 0.05 to 0.62. Subsequently, we elucidated that the moisture and nutritional content in the egg yolk were not influenced by the percentage of yolk but were indeed subject to age regulation. There was a notable decrease in moisture, an elevation in crude fat, and an increase in the diversity of fatty acids of yolk with advancing age. In summary, investigating the trends and interrelationships in egg quality, nutrient content, and heritability across the whole laying cycle offers valuable insights for breeders to optimize feeding management strategies and aids consumers in meeting their expectations of egg quality.

## 1. Introduction

Eggs occupy a prominent position within the global food market, representing a cost-effective and readily accessible source of dietary protein [1]. The substantial protein furnishes essential nutrients for cellular structure, repair processes, immune system regulation, and metabolic balance [2]. At the same time, the vitamins, minerals, and antioxidants present in eggs provide comprehensive health protection, significantly contributing to the maintenance of human health [3,4]. Ensuring egg quality holds paramount significance for human health and producer economic benefits. Egg qualities comprise internal attributes such as yolk weight (YW), yolk color (YC), percentage of yolk (PY), albumen height (AH), haugh unit (HU), and external characteristics including egg weight (EW), eggshell weight (ESW), eggshell color (ESC), eggshell strength (ESS), eggshell index (ESI), and eggshell thickness (EST).

Numerous studies have elucidated the primary factors influencing egg quality, including genetics and environmental conditions. Madalena Lordelo et al. found that four native chicken breeds in Portugal, while exhibiting lower egg weights compared to hybrid breeds, demonstrated superior egg shape and HU [5]. Zhang et al. found that by adding different qualities and concentrations of vegetable oil and lard to the diet, vegetable oil could effectively enhance the quality of eggs. Conversely, oxidized lard not only reduced egg quality but also significantly altered the microstructure of the egg yolk [6]. Nathaniel W. Barrett et al. observed a significant change in EW between the first two weeks and the latter two weeks of heat stress [7]. Indeed, apart from those factors, the age of the hen is also a significant determinant affecting egg quality. Adam Kraus et al. revealed a gradual increase in EW and a decrease in EST and ESS with age by using Czech golden spotted hens, which were fed in cages [8]. J. Vlčková et al. investigated significant differences in EW, HU, and albumen pH between 26 and 51 weeks of age [9]. F. Sirri et al. investigated the egg quality changes in commercial poultry from 31 to 81 weeks, in which EW and egg surface area increased significantly [10].

Genetic parameters related to egg quality have consistently garnered attention in research. Ongoing progress in this domain substantially contributes to refining breeding methodologies and elevating egg quality. Zhang et al. conducted an assessment of the heritability of egg quality traits, including EW (0.63), ESI (0.40), ESS (0.24), EST (0.34), ESC (0.46), and ESW (0.64) in 920 brown-egg dwarf layers originating from 44 sires [11]. Alipanah et al. estimated the heritability of various egg quality traits in zhazak layers, and found that the AH, ESC, YC, ESW, EW, HU, and YW were 0.42, 0.15, 0.19, 0.54, 0.50, 0.46, and 0.32, respectively [12]. Bécot et al. calculated that the heritability of EW, ESC, ESS, and AH from Rhode Island Red and White Leghorn hens to be 0.45 and 0.46, 0.37 and 0.17, 0.24 and 0.16, 0.40 and 0.23, respectively [13].

In summary, the materials of the current related research often focus on the western introduced breeds with higher market shares. The quality of eggs is typically assessed based solely on external and internal factors, overlooking the inherent nutritional components of the eggs. Therefore, this study focused on Wenchang chickens of native breed and investigated the evolving egg qualities, heritability, and phenotypic correlation of egg quality traits, yolk moisture, and nutritional components throughout the entire laying cycle. The objective was to enhance our comprehension of the dynamic changes in egg qualities for producing high-quality egg products and meeting market demands.

## 2. Materials and Methods

### 2.1. Experimental Design and Diets

**Figure 1:**
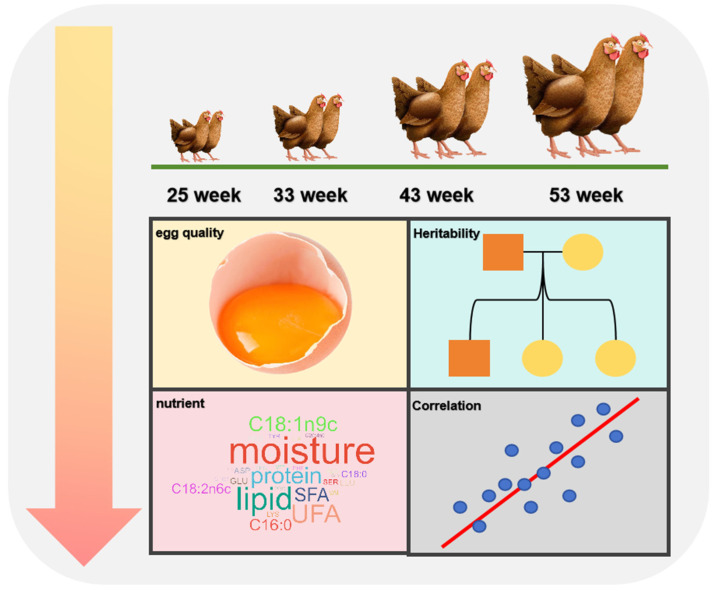
Study workflow of the present study.

In this study, we employed the Chinese native breed of hens, known as the Wenchang chicken, the only indigenous chicken breed listed in the ‘animal genetic resources in China (poultry)’ in Hainan province. It is famous for its excellent meat quality, especially its thin skin, tenderness and juiciness, and is sold all over southeast Asia [14]. There are few studies on the egg quality traits. Therefore, a total of 531 healthy hens from 20 sires were selected based on the body size, health status, and production performance at 25 week. The performance and body weight of each laying hen was monitored and eggs were collected on a daily basis. Then, we conducted measurements and analyses on egg qualities, heritability, and correlation of egg quality traits and yolk nutritional components at 33, 43, and 53 weeks (Figure 1). Each time point was 6 experimental days, in which the initial 3 days were used to assess egg qualities, and the subsequent 3 days were used to collect yolk for detecting moisture and nutrients.

All hens were housed individually in cages with an area of 500 cm^2^ under a light-dark cycle of 16 h light and 8 h dark (16L:8D), equipped with an individual feeder and water. The ingredients of the diets are shown in Table 1.

### 2.2. The Detection of Egg Quality

Egg qualities are the crucial aspect of poultry production. This study focused on calculating egg quality parameters, specifically ESI, ESS, EW, AH, YC, HU, YW, ESW, EST, and PY at 3 distinct time points: 33, 43, and 53 weeks. We collected 587 eggs (33 week), 784 eggs (43 week), and 284 eggs (53 week) in the initial 3 days, and all egg quality indicators were assessed on the day of collection.

The modified ESI was employed according to previous research and calculated as ESI = egg length/egg width [15]. The ESS was measured using the Model-II Eggshell Strength Tester (Robotmation, Tokyo, Japan). The EMT5200 Multifunctional Egg Tester (Robotmation, Tokyo, Japan) facilitated the acquisition of EW, AH, YC, and HU. The following is the HU equation:

HU = 100 log(H + 7.57 − 1.7 W^0.37^), (1)

where H denotes AH (mm) and W represents EW (g) [16,17]. Yolks were separated from the contents of the eggs, and YW was obtained by P601N electronic balance (Qinghai Corporation, Shanghai, China). Additionally, the ESW was determined by weighing the shell after drying and membrane removal using an YP601N electronic balance (Qinghai Corporation, Shanghai, China). The EST was evaluated in 3 areas (at both ends and the equator), and the average of 3 measurements was recorded. Finally, the PY was obtained using the formula:

PY = YW/EW,(2)

We organized all the egg qualities at different time points using Excel. We performed one-way Analysis of Variance and Duncan’s multiple range test with time as a factor using the Statistical Package for the Social Sciences (SPSS) 25. A significance level of *p* < 0.05 indicated statistically significant differences, while a significance level of *p* < 0.01 denoted highly statistically significant differences [18]. Then, we calculated the Pearson correlation between different egg qualities with all phenotypic data and visualized the heatmap with the ‘corrplot’ package.

### 2.3. The Relationship of Body Weight and Onset of Production with Egg Quality

Based on the production records for the hens, we obtained the onset of production and body weight at 43 weeks for each hen. And, the Spearman’s correlations between onset of production and egg qualities, body weight, and egg qualities were calculated with ‘cor’ function in R language and mapped using the ggplot2 package. The statistically derived *p* < 0.05 indicated the presence of a significant correlation.

### 2.4. The Detection of Yolk Moisture and Nutritional Components

Based on the egg quality results from Section 2.2 and the breed’s performance, in order to explore the differences in moisture and nutrient content of yolks with different PY, we aimed to collect 3 consecutive egg yolks produced by each hen during the last 3 days. The 3 yolks from the same hen were mixed to form one sample as a potential test sample. Therefore, during the yolk collection over the last 3 days, we exclusively focused on collecting yolks from the top 18% and bottom 18% ranked hens based on PY rankings. Ultimately, for the purpose of conducting moisture and nutrient analysis, ten hens’ yolks were selected at each time point due to the influence of both EW and YW on PY, and five with significantly higher PY and 5 with significantly lower PY but with no significant difference in EW (Table S1). The yolks, carefully extracted, were preserved at a sub-zero temperature of −20 °C daily.

We compared differences in moisture, protein, crude fat, saturated fatty acid (SFA), monounsaturated fatty acid (MUFA), and polyunsaturated fatty acid (PUFA) across different PY at same time point. Subsequently, we compared differences in moisture, protein, amino acids, crude fat, SFA, MUFA, and PUFA at different time points. The method used to identify significant differences was the *t*-test [19].

The methods used for the analysis of moisture and various nutritional components are as follows.

Moisture Analysis: The assessment of moisture was first performed using the GB 5009.3-2016 method. Utilizing the physical properties of moisture in yolk, the loss on drying method is employed to measure the weight loss of samples at a temperature range of 101 °C to 105 °C and a pressure of 101.3 kPa (equivalent to atmospheric pressure). This method accounts for the weight loss attributed to absorbed moisture, partially bound water, and substances that can volatilize under these conditions. The moisture content is then calculated based on the difference in weight before and after drying

Protein Analysis: The determination of protein content was first conducted using the GB 5009.5-2016 method. In yolk, proteins undergo decomposition under catalytic heating conditions, generating ammonia that combines with sulfuric acid to produce ammonium sulfate. Alkaline distillation liberates the ammonia, which is then absorbed using boric acid and titrated with standard sulfuric acid or hydrochloric acid solution. The nitrogen content is determined based on the amount of acid consumed and multiplied by a conversion factor to ascertain the protein content.

Crude fat Analysis: The measurement of crude fat content was carried out using the the second method of the GB 5009.6-2016 method. The bound fats in a yolk need to be liberated using a strong acid to render them free, and these liberated fats are easily soluble in organic solvents. After the sample undergoes hydrolysis with hydrochloric acid, extraction using anhydrous ether or petroleum ether is employed. Removing the solvent yields the total content of free and bound fats.

Amino Acid Analysis: Eighteen amino acids, including aspartic acid (ASP), threonine (THR), serine (SER), glutamic acid (GLU), glycine (GLY), Alanine (ALA), valine (VAL), methionine (MET), isoleucine (ILE), leucine (LEU), tyrosine (TYR), phenylalanine (PHE), lysine (LYS), histidine (HIS), arginine (ARG), and proline (PRO), except for tryptophan (TRP) and cysteine (CYS), which were determined using an amino acid analyzer, were analyzed using the GB 5009.124-2016 method. In yolk, proteins are hydrolyzed into free amino acids by hydrochloric acid. After separation using an ion exchange column, they react with ninhydrin solution, generating color reactions. The amino acid content is then determined using a visible spectrophotometric detector.

Fatty Acid Analysis: The fatty acids were calculated using the the first way of GB 5009.168-2016 method. After the fats in the sample that have been combined with internal standard are extracted with hydrolysis-ether solution, the sample is saponified and methyl-esterified under alkaline conditions to produce fatty acid methyl esters. After analysis using capillary column gas chromatography, the internal standard method is used to quantitatively determine the content of fatty acid methyl esters. According to the content and conversion coefficients of various fatty acid methyl esters, calculate the content of fatty acids.

The details of all test methods are given in Supplementary S1. All of the above tests were repeated twice, and the final value was the average of the two values.

### 2.5. The Estimation of Heritability of Egg Quality

HiBLUP software (Version: 1.3.1 Wuhan, Hubei, China) was used to estimate the variance and covariance components with pedigree and phenotype information of the hens (Table S2) [20]. An animal threshold model was used to analyze the heritability of EW, YW, PY, ESW, PSW, ESS, EST, and ESI.

The animal model was constructed as follows:

y = Xβ + Za + e,(3)

where y represents the vector of phenotypic value, β represents the vector of “fixed” effects, a represents the vector of random additive genetic effects of all individuals, e represents the vector of random residuals, and X and Z are appropriate correlation matrices. The average information restricted maximum likelihood (AIREML) algorithm in Hiblup software was used for estimating variance components of traits.

## 3. Results

### 3.1. Pattern of Egg Quality

In this study, we compared multiple egg qualities across different weeks of age and observed a significant increase in EW, YW, PY, and ESI with age (Table 2). However, we found that there was a more significant growth rate between 33 and 43 weeks compared to the growth rate between weeks 43 and 53. Additionally, we observed that parameters like ESW (4.90 g), ESS (kg/cm^2^), and EST (315.27 μm) were highest at 33 weeks compared to the other time points, and it was evident that there was a decrease in eggshell quality with age. However, with an increase in both EW (33 weeks: 46.10 g, 43 weeks: 48.74 g, 53 weeks: 49.50 g) and YW (33 weeks: 13.36 g, 43 weeks: 15.08 g, 53 weeks: 15.50 g), there was also a gradual rise in PY (33 weeks: 0.29, 43 weeks: 0.31, 53 weeks: 0.31), which suggested that the deposition capacity of yolk gradually increased with age. And, we found that there was no further change in PY between weeks 43 and 53. Lastly, in terms of the HU, an indicator of egg freshness, we observed the highest value at 33 weeks, and it was significantly higher than the values in other weeks (*p* < 0.05). These findings revealed the dynamic changes in egg quality and provided insights into the physiological development of this breed.

### 3.2. Correlation of Egg Quality

**Figure 2:**
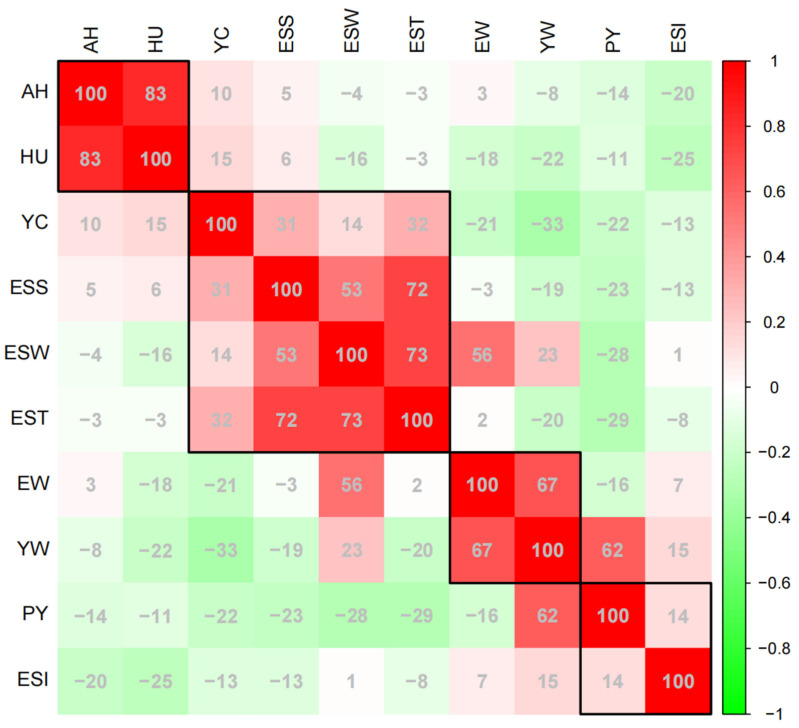
Correlation between different egg quality traits. A numerical range of “−100”−“100” corresponds to a correlation scale from “−1”−“1”. Egg weight (EW), eggshell strength (ESS), yolk weight (YW), yolk color (YC), eggshell weight (ESW), egg shape index (ESI), eggshell thickness (EST), percentage of yolk (PY), haugh unit (HU), and albumen height (AH). The correlation of traits in the black squares is stronger.

To further evaluate the Pearson correlation coefficient between various traits, we combined the egg quality data from different weeks (Figure 2). Among of these, AH and HU had a strong positive correlation, and their correlation coefficient was as high as 0.83. ESS, ESW, and EST were mutually influenced and displayed a consistently strong positive correlation among themselves. Meanwhile, we found that YC was positively correlated with both ESS and EST, and the correlation indexes were 0.31 and 0.32, respectively, while YC was negatively correlated with YW, and the correlation index was −0.33. In addition, the positive correlation between EW and YW was higher than that between EW and ESW, the PY was only affected by YW, and the correlation index between them was as high as 0.62. Furthermore, the correlation between traits at different time points can be found in Figure S1.

### 3.3. Effect of Onset of Production and Body Weight on Egg Quality

**Figure 3:**
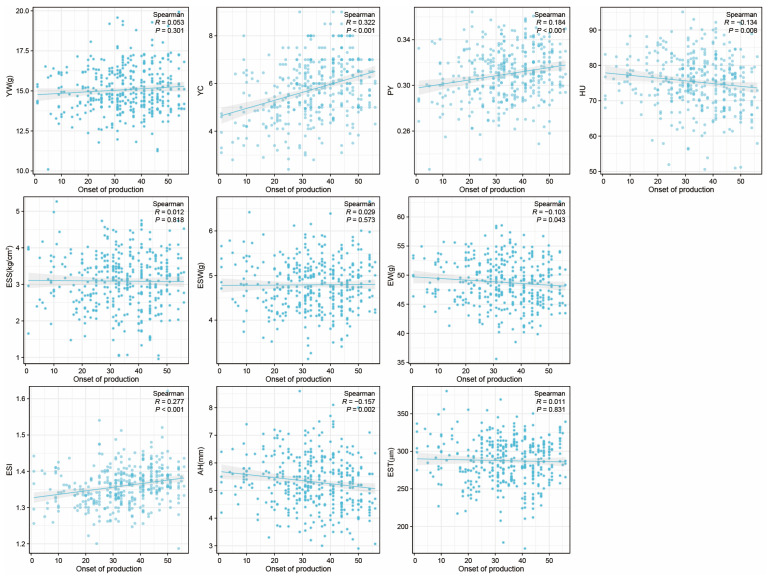
The correlation between onset of production and egg qualities at 43 weeks. X-axis represents the ‘onset of production’ with the first day of the first egg production recorded as 1, and so on. In this study, the onset of production ranges from 1 to 56, where values closer to 1 indicate an earlier onset of production. Egg weight (EW), eggshell strength (ESS), yolk weight (YW), yolk color (YC), eggshell weight (ESW), egg shape index (ESI), eggshell thickness (EST), percentage of yolk (PY), haugh unit (HU), and albumen height (AH).

In this study, we investigated the Spearman correlation coefficient between the onset of egg production and egg qualities at 43 week. The results indicate that the onset of production is negatively correlated with EW, AH, and HU (*p* < 0.05), positively correlated with PY, YC, and ESI (*p* < 0.05), and had no significant correlation with other YW, ESS, EST, and ESW (Figure 3). We found that among all the traits, YC showed the strongest correlation with the onset of production, and it was positively correlated (R = 0.322).

**Figure 4:**
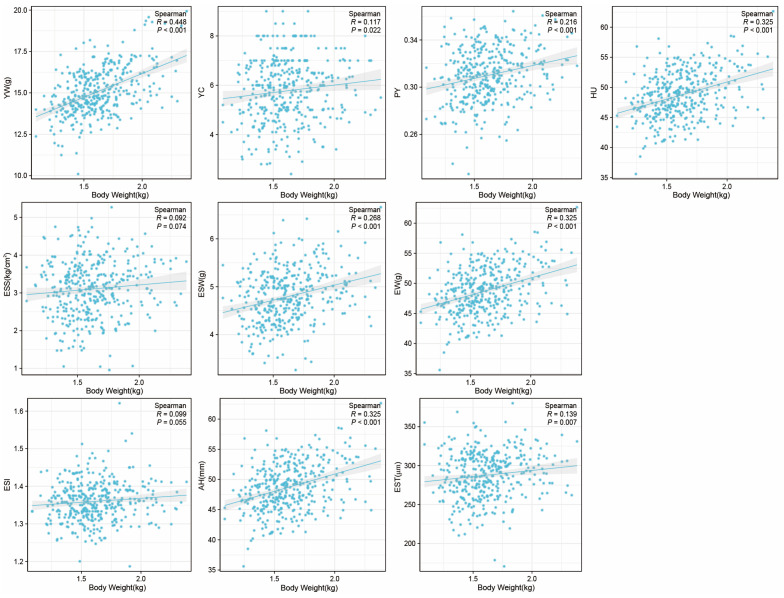
The correlation between body weight and egg qualities at 43 week. X-axis represents the body weight at 43 weeks. Egg weight (EW), eggshell strength (ESS), yolk weight (YW), yolk color (YC), eggshell weight (ESW), egg shape index (ESI), eggshell thickness (EST), percentage of yolk (PY), haugh unit (HU), and albumen height (AH).

Furthermore, we also investigated the impact of body weight on egg qualities, and it was found to have a positive correlation with EW, PY, HU, YW, YC, EST, ESW, and AH, while no significant correlation was observed with ESS and ESI (Figure 4).

### 3.4. Estimation of Genetic Heritability for Egg Quality

**Figure 5:**
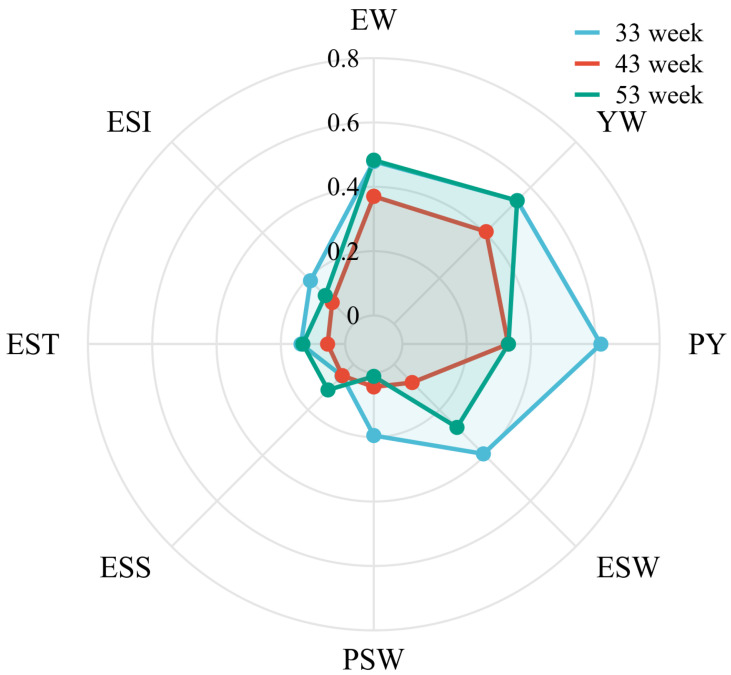
Heritability of different egg quality traits. Egg weight (EW), eggshell strength (ESS), yolk weight (YW), yolk color (YC), eggshell weight (ESW), egg shape index (ESI), eggshell thickness (EST), percentage of yolk (PY), haugh unit (HU), and albumen height (AH).

Seven phenotypic traits were analyzed for heritability (Figure 5). Most of the traits showed moderate to high heritability, including EW (33 weeks: 0.48, 43 weeks: 0.37, 53 weeks: 0.48), YW (33 weeks: 0.54, 43 weeks: 0.41, 53 weeks: 0.54), PY (33 weeks: 0.62, 43 weeks: 0.33, 53 weeks: 0.33), and ESW (33 weeks: 0.39, 43 weeks: 0.08, 53 weeks: 0.28). There were also some traits with low heritability, including ESS (33 weeks: 0.05, 43 weeks: 0.05, 53 weeks: 0.11), EST (33 weeks: 0.14, 43 weeks: 0.05, 53 weeks: 0.13), ESI (33 weeks: 0.19, 43 weeks: 0.09, 53 weeks: 0.12).

### 3.5. Comparison of Moisture and Nutritional Components with Different Percentage of Yolk

**Figure 6:**
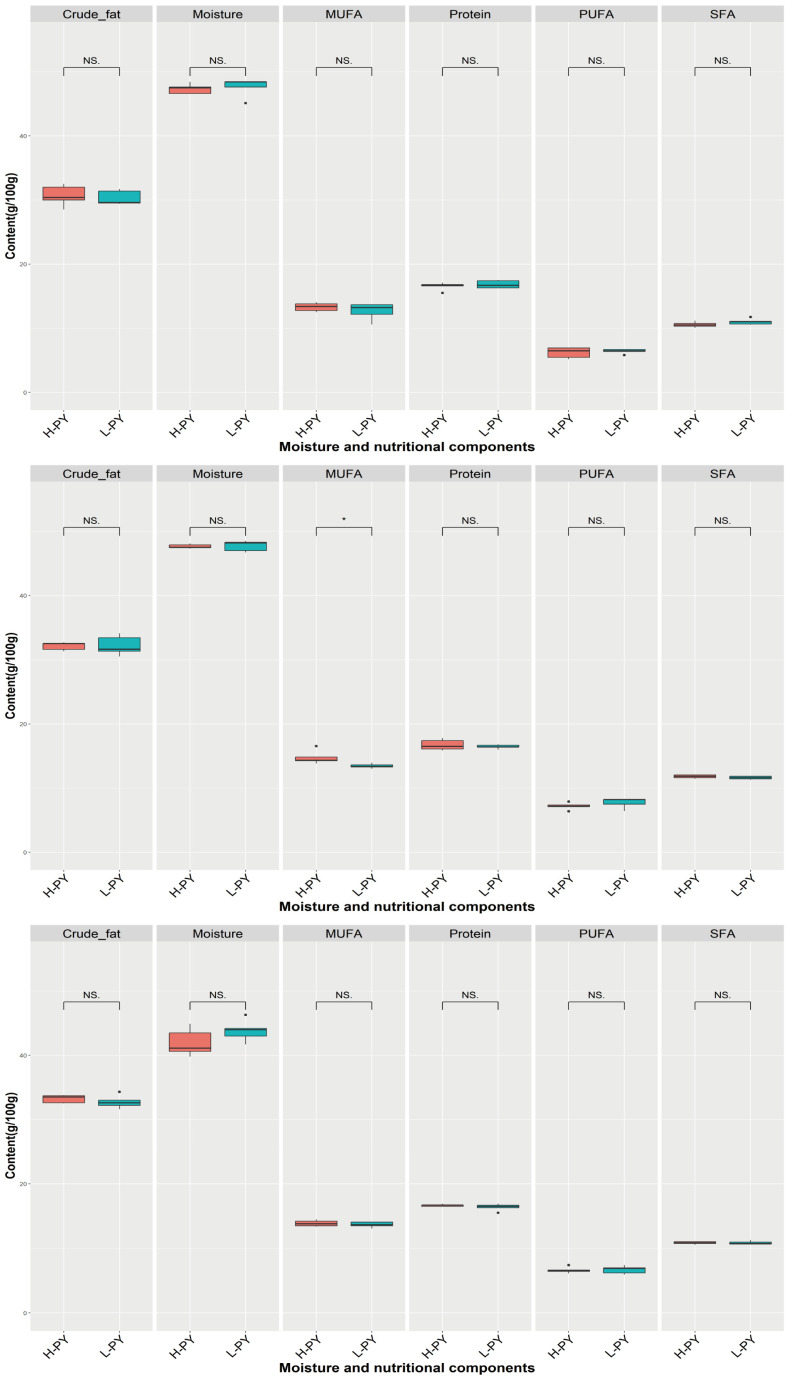
Comparison of moisture and nutritional components under different PY at 33, 43, and 53 weeks. H-PY means high percentage of yolk and color is red, L-PY means low percentage of yolk and color is blue. The 3 figures from top to bottom represent 33, 43, and 53 weeks, respectively. * denotes *p* < 0.05, NS denotes no significant difference. Saturated fatty acid (SFA), monounsaturated fatty acid (MUFA), and polyunsaturated fatty acid (PUFA).

In our study, we observed that the primary contents in the yolk were moisture, followed by crude fat, proteins, MUFAs, SFAs, and PUFAs. However, except for significantly higher MUFA in high-PY than in low-PY at 43 weeks, no significant differences were observed in other cases (Figure 6). And the range of moisture, crude fat, proteins, MUFAs, SFAs, and PUFAs were 39.8 g/100 g–48.5 g/100 g (average: 46.03 g/100 g), 28.5 g/100 g–34.3 g/100 g (average: 31.88 g/100 g), 15.5 g/100 g–17.8 g/100 g (average: 16.60 g/100 g), 10.6 g/100 g–16.6 g/100 g (average: 13.63 g/100 g), 10.1 g/100 g–12.2 g/100 g (average: 11.12 g/100 g) and 5.2 g/100 g–8.3 g/100 g (average: 6.81 g/100 g).

### 3.6. Comparison of Moisture and Nutritional Components across Different Weeks

**Figure 7:**
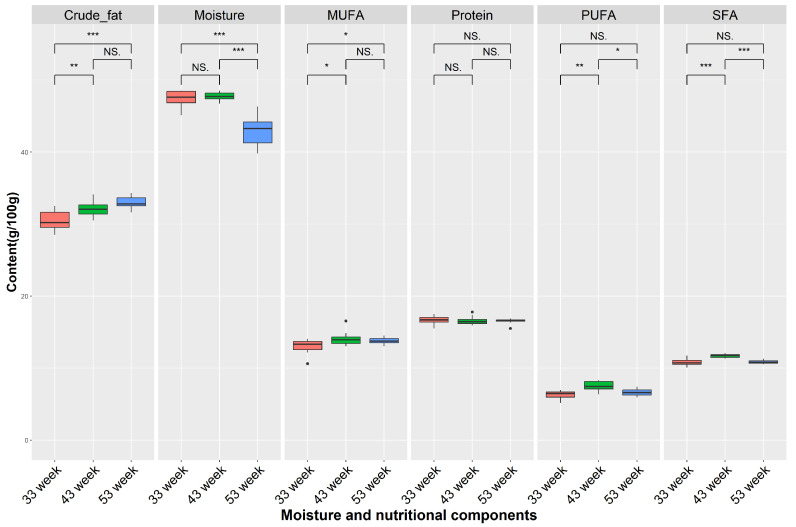
Comparison of moisture and nutritional components under different time points. NS denotes *p* > 0.05, * denotes *p* < 0.05, ** denotes *p* < 0.01, *** denotes *p* < 0.001, NS denotes no significant difference. Saturated fatty acid (SFA), monounsaturated fatty acid (MUFA), and polyunsaturated fatty acid (PUFA). Red box means 33 week, green box means 43 week, blue box means 53 week.

Firstly, we compared the moisture, crude fat, protein, SFAs, MUFAs, and PUFAs based on the different weeks (Figure 7), which revealed a significant decrease in moisture and a significant increase in crude fat content in the egg yolks at 53 weeks compared to at 33 weeks (*p* < 0.001). And, we found that the content of MUFAs at 33 weeks was significantly lower than other weeks (*p* < 0.05), while the content of PUFAs (7.46 g/100 g) and SFAs (11.85 g/100 g) at 43 weeks were the highest.

**Figure 8:**
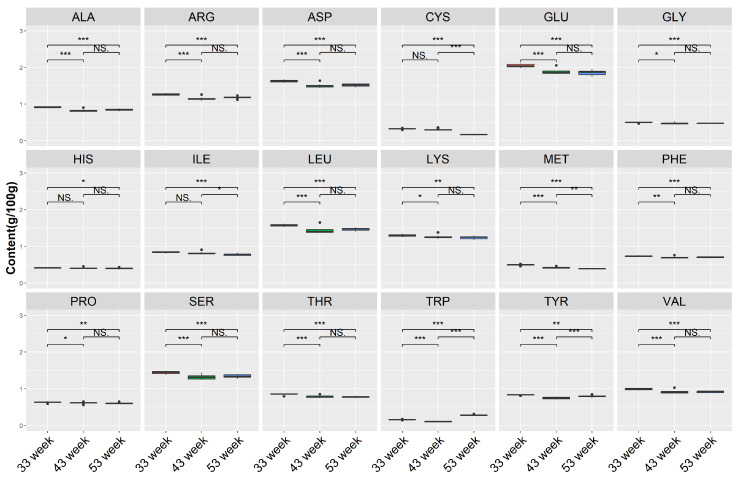
Comparison of amino acids under different time points. NS denotes *p* > 0.05, * denotes *p* < 0.05, ** denotes *p* < 0.01, *** denotes *p* < 0.001. Aspartic acid (ASP), threonine (THR), serine (SER), glutamic acid (GLU), glycine (GLY), Alanine (ALA), valine (VAL), methionine (MET), isoleucine (ILE), leucine (LEU), tyrosine (TYR), phenylalanine (PHE), lysine (LYS), histidine (HIS), arginine (ARG), proline (PRO), tryptophan (TRP), and cysteine (CYS). Red box means 33 week, green box means 43 week, blue box means 53 week.

Moreover, we detected a change in amino acids among different weeks (Figure 8), among the 18 amino acids, and the top 5 in terms of content were GLU, ASP, LEU, SER, and LYS. The ALA, ARG, ASP, GLU, GLY, LEU, LYS, PHE, PRO, SER, THR, and VAL content at 33 weeks was significantly higher than other weeks, and there were no differences between weeks 43 and 53. Meanwhile, the TRP at 53 weeks was significantly higher than other weeks.

Finally, the fatty acids which were identified are shown in Table 3, including myristic acid (C14:0), myristoleic acid (C14:1n5), pentadecylic acid (C15:0), palmitic acid (C16:0), palmitoleic acid (C16:1n7), margaric acid (C17:0), cis-10-Heptadecenoic acid (C17:1n7), stearic acid (C18:0), oleic acid (C18:1n9c), linoleic acid (C18:2n6c), γ-Linolenic acid (C18:3n6), arachidic acid (C20:0), cis-11-Eicosenoic acid (C20:1), all cis-11,14 -Eicosadienoic acid (C20:2), behenic acid (C22:0), dihomo-γ-linolenoic acid (C20:3n6), arachidonic acid (C20:4n6), lignoceric acid (C24:0), nervonic acid(C24:1), and all cis-4,7,10,13,16,19-Docosahexaenoic acid (C22:6n3). The variety of fatty acids in the yolk continued to expand with increasing age, in which C20:0, C22:0, C24:0, and C24:0 were only detected at 53 weeks. And the contents of C16:0 and C18:0 at 43 weeks were significantly higher than that at 33 and 43 weeks (*p* < 0.01).

## 4. Discussion

### 4.1. Egg Quality

In our study, EW, YW, and PY significantly increased with age (Table 2), and were reasons for prolonged duration of rearing, resulting in an increase in body weight, which was significantly positively correlated with EW [21,22,23]. However, it was observed that the rate of increase slowed down between weeks 43 and 53, which is due to the native breed of hens we selected, with a relatively shorter duration compared to Western introduced breeds. At 53 weeks, hens enter the later stage of egg production, with reduced feed conversion efficiency and decreased production performance, leading to a decrease in the growth rate [24,25]. Furthermore, a significant decrease in ESW and ESS was observed at 43 weeks, which might be attributed to the reduced consumption of nutrients during the formation of eggshells in order to meet the nutritional demands of egg production at peak laying. Hence, calcium trace elements and other or minerals may be suitably supplemented into the feed during the late phase of egg production to enhance the quality of eggshells [26]. The ESI and HU showed significant changes, which were significantly correlated with consumer preferences, between the early and late stages of egg production [27]. Because consumers generally prefer to purchase eggs with a more oval shape according to market research [28], and a higher HU value indicates better egg quality, elevating the HU value can increase the viscosity of the albumen [29], ensuring sufficient bioactive substances in the albumen [30] and making eggs more appealing to consumers. Egg yolk color was lighter at 43 and 53 weeks, and from the perspective of yolk color, consumers were more inclined to eggs of 33 weeks [31]. Therefore, with the aging of hens, breeders should consider improving the ESI and enhancing HU and YC to better attract consumers and increase their own economic benefits.

Correlation analysis of egg quality can provide producers with a better understanding different egg quality characteristics [32]. In this study, a strong positive correlation was found between AH and HU, EW and YW, as well as positive correlations among ESS, EST, and ESW, which is consistent with previous studies [11,33]. Meanwhile, YC had negative correlation with YW and EW, and HU had a negative correlation with ESI (Figure 2). Therefore, breeders should balance breeding in the breeding process and not blindly select hens with a high EW for breeding, as this might compromise the quality of YC. Consumers can identify fresher eggs by selecting those with a rounder shape when making purchases.

Finally, this study found that the hens which achieved maturity earlier produced eggs with a greater HU, and EW and YW were opposite (Figure 3). This suggests that hens started laying at a relatively younger age tend to produce smaller eggs but with better freshness. The earlier laying hens had a weaker ability to deposit yolk. On the other hand, investigating of the correlation between body weight and egg qualities at 43 weeks suggests that, while ensuring hens’ health, appropriately increasing body weight contributed to improving egg qualities.

### 4.2. Heritability Estimates

In the field of egg quality research, the evaluation of egg quality heritability holds significant importance for both the food industry and agricultural production [4]. Traits such as EW, YW, PY, ESW, ESS, EST, and ESI reflect the quality and morphological characteristics, as well as the stability and safety during production. In this study, EW, YW, PY and ESW exhibit high heritability, aligning with previous findings [11,12,34], implying that genetic factors play a more substantial role in determining these four traits. Conversely, the heritability of ESI, ESS, and EST were relatively low, which was also consistent with previous studies [11,12,34], meaning that the premise of maintaining the consistency of eggshell quality is to decrease the effect of environment. In conclusion, the evaluation of genetic parameters furnishes a solid scientific foundation for enhancing the quality, safety, and sustainability of eggs. It is essential to comprehensively evaluate the heritability of various egg quality traits and optimize the selection and pairing of individuals to ensure that subsequent generations produce eggs of elevated quality.

### 4.3. Moisture and Nutritional Components

Eggs, as a nutritionally rich food, including protein, MUFAs, SFAs, PUFAs, and essential amino acids, contribute to maintaining bodily health and providing abundant energy [35]. In this study, we investigated the main components of yolks at 33, 43, and 53 weeks under different PY, including moisture, protein, crude fat, SFA, MUFA, and PUFA. Overall, there were no significant differences observed except for a significant difference in MUFA at 43 weeks (*p* < 0.05), indicating that varying PY did not significantly impact the overall nutritional value of the yolk.

Furthermore, we examined the changes in the main component contents in yolks at different time points. The study showed a significant decrease in moisture content and a significant increase in the crude fat content of yolks during the later stage of egg production. These changes might be related to the physiological status of the hens and increased feed intake at the end of the egg production cycle [36,37]. The metabolic capacity of organisms decreases during this stage, leading to a decrease in moisture content and an increase in crude fat content during yolk deposition [38].

The levels of MUFAs and PUFAs at 43 weeks were significantly higher than those at 33 and 53 weeks. It has been shown that unsaturated fatty acid in yolks contributes to the self-regulation of human cholesterol intake [39]. Therefore, consumers with a standard daily cholesterol intake should be more inclined to buy mid-laying eggs.

Through the analysis of amino acids in yolks, we found that most amino acids had the highest content during the early stage of egg production. Although statistically significant differences were observed, the content of various amino acids was not very high, and the total protein content showed no significant differences. Thus, consumers can rest assured when purchasing eggs, as eggs remain a high-quality source of animal protein intake at any time.

Finally, our analysis of various fatty acids revealed that as the age of the hen increased, the variety of fatty acids in the yolk became more diverse. During the later stage of egg laying, we identified C20:0, C22:0, C24:0, and C24:0, even though their quantities were low and had not been previously identified. Interestingly, we found that the newly identified fatty acids during the later stage of egg laying were all saturated fatty acids. Studies have indicated that an increased intake of saturated fatty acids may lead to elevated levels of low-density lipoprotein cholesterol in the blood [40], significantly correlating with a higher risk of cardiovascular disease [41,42]. Therefore, poultry farmers should promptly eliminate hens during the breeding process to provide the consumer market with high-quality eggs.

## 5. Conclusions

In conclusion, several aspects of egg quality, including EW, YW, PY, and ESI significantly increased, while HU declined with aging. The earlier onset of production was beneficial for increasing EW and HU, and body weight exhibited a positive correlation with the majority of egg qualities. Regarding heritability, the traits exhibited heritability values ranging from 0.05 to 0.62. Moreover, the moisture, crude fat, c16:0, c18:0, and most amino acids were significantly altered at different time points. Our findings offer a novel perspective for the investigation of egg quality, patterns of change, and correlations influencing various factors and for evaluating of heritability for traits throughout the entire laying cycle of native breed.

## Figures and Tables

**Table 1:** Composition of hens’ diet.

Item	Ingredient, %
Corn	61.95
Soybean meal	24.53
Soy oil	1.00
Stone powder	8.50
Premix ^1^	3.00
Zeolite powder	1.00
Antioxidant ^2^	0.02
Total	100.00

^1^ Premix: Mineral premix provided per kilogram (kg) of diet: Mn, 100 mg; Fe, 80 mg; Zn, 75 mg; Cu, 8 mg; I, 0.35 mg; Se, 0.15 mg. Vitamin premix provided per kg of diet: vitamin A, 12,500 International units (IU); vitamin D3, 2500 IU; vitamin E, 30 IU; vitamin K3, 2.65 mg; vitamin B1, 2 mg; vitamin B2, 6 mg; vitamin B12, 0.025 mg; biotin, 0.0325 mg; folic acid, 1.25 mg; pantothenic acid, 12 mg; niacin, 50 mg. ^2^ Antioxidant = ethoxyquin.

**Table 2:** Egg quality at different weeks of the production cycle ^1^.

Item ^2^	33 Week	43 Week	53 Week
EW, g	46.10 ± 3.70 ^Bb^	48.74 ± 3.91 ^a^	49.50 ± 4.41 ^A^
YW, g	13.36 ± 1.21 ^C^	15.08 ± 1.42 ^B^	15.50 ± 1.32 ^A^
PY	0.29 ± 0.02 ^B^	0.31 ± 0.02 ^A^	0.31 ± 0.02 ^A^
ESW, g	4.90 ± 0.46 ^a^	4.79 ± 0.56 ^b^	4.88 ± 0.65
ESS, kg/cm^2^	3.65 ± 0.62 ^A^	3.09 ± 0.75 ^B^	3.03 ± 0.85 ^B^
EST, μm	315.27 ± 22.49 ^A^	287.56 ± 30.28 ^C^	295.50 ± 39.13 ^B^
ESI	1.35 ± 0.05 ^C^	1.36 ± 0.05 ^B^	1.39 ± 0.06 ^A^
AH, mm	5.87 ± 1.25 ^A^	5.32 ± 0.95 ^Bb^	5.57 ± 0.92 ^aB^
YC	8.06 ± 0.90 ^A^	5.76 ± 1.26 ^B^	4.59 ± 1.20 ^C^
HU	80.04 ± 6.30 ^A^	75.35 ± 7.48 ^Bb^	76.99 ± 7.01 ^aB^

^1^ Each value is presented as mean ± standard deviation. In the same row, different uppercase letters in the superscript indicate highly significant differences (*p* < 0.01), the same uppercase letters indicate no highly significant difference (*p* > 0.01), while different lowercase letters indicate significant differences (*p* < 0.05), and the same lowercase letters or no letters indicate no significant differences (*p* > 0.05), as analyzed by one-way ANOVA. ^2^ egg weight (EW), eggshell strength (ESS), yolk weight (YW), yolk color (YC), eggshell weight (ESW), egg shape index (ESI), eggshell thickness (EST), percentage of yolk (PY), haugh unit (HU), and albumen height (AH).

**Table 3:** The content of fatty acids of yolk at different weeks ^1^.

Item ^2^	33 Week	43 Week	53 Week
C14:0, g/100 g	0.144 ± 0.122	0.103 ± 0.349	0.100 ± 0.008
C14:1n5, g/100 g	0.017 ± 0.007	0.026 ± 0.033	0.021 ± 0.005
C15:0, g/100 g	0.017 ± 0.012	0.018 ± 0.003	0.018 ± 0.003
C16:0, g/100 g	7.792 ± 0.391 ^B^	8.496 ± 0.235 ^A^	8.213 ± 0.308 ^B^
C16:1n7, g/100 g	0.755 ± 0.136	0.806 ± 0.136	0.782 ± 0.133
C17:0, g/100 g	0.056 ± 0.009 ^a^	0.056 ± 0.006 ^A^	0.050 ± 0.008 ^Bb^
C17:1n7, g/100 g	0.022 ± 0.005	0.024 ± 0.007	0.024 ± 0.002
C18:0, g/100 g	2.793 ± 0.165 ^B^	3.080 ± 0.162 ^A^	2.904 ± 0.171 ^B^
C18:1n9c, g/100 g	12.145 ± 0.987	13.190 ± 1.100	13.044 ± 0.492
C18:2n6c, g/100 g	5.417 ± 0.567	6.032 ± 0.673	5.708 ± 0.467
C18:3n3, g/100 g	0.128 ± 0.025 ^b^	0.161 ± 0.031 ^a^	0.145 ± 0.016 ^b^
C18:3n6, g/100 g	0.035 ± 0.010	0.036 ± 0.006	0.035 ± 0.007
C20:0, g/100 g	-	-	0.008 ± 0.001
C20:1, g/100 g	0.054 ± 0.016	0.067 ± 0.106	0.066 ± 0.009
C20:2, g/100 g	-	0.060 ± 0.011	0.056 ± 0.007
C20:3n6, g/100 g	0.050 ± 0.007	0.050 ± 0.009	0.051 ± 0.009
C20:4n6, g/100 g	0.734 ± 0.097	0.768 ± 0.047	0.733 ± 0.053
C22:0, g/100 g	-	-	0.018 ± 0.004
C24:0, g/100 g	-	-	0.011 ± 0.002
C24:1, g/100 g	-	-	0.016 ± 0.002
C22:6n3, g/100 g	-	0.360 ± 0.041	0.335 ± 0.028

^1^ Each value is presented as mean ± standard deviation. In the same row, different uppercase letters in the superscript indicate highly significant differences (*p* < 0.01), the same uppercase letters indicate no highly significant difference (*p* > 0.01), while different lowercase letters indicate significant differences (*p* < 0.05), and the same lowercase letters or no letters indicate no significant differences (*p* > 0.05). ^2^ myristic acid (C14:0), myristoleic acid (C14:1n5), pentadecylic acid (C15:0), palmitic acid (C16:0), palmitoleic acid (C16:1n7), margaric acid (C17:0), cis-10-Heptadecenoic acid (C17:1n7), stearic acid (C18:0), oleic acid (C18:1n9c), linoleic acid (C18:2n6c), γ-Linolenic acid (C18:3n6), arachidic acid (C20:0), cis-11-Eicosenoic acid (C20:1), all cis-11,14 -Eicosadienoic acid (C20:2), behenic acid (C22:0), dihomo-γ-linolenoic acid (C20:3n6), arachidonic acid (C20:4n6), lignoceric acid (C24:0), nervonic acid(C24:1), all cis-4,7,10,13,16,19-Docosahexaenoic acid (C22:6n3).

## Data Availability

The data used to support the findings of this study can be made available by the corresponding author upon request.

## Supplementary Materials

The following supporting information can be downloaded at: https://www.mdpi.com/article/10.3390/foods12244472/s1, Figure S1: The correlation of the egg qualities at different weeks; Table S1: The EW, YW and PY of chosen hens at different weeks. Table S2: The pedigree and phenotype of hens. Supplementary S1: Detailed test methods for moisture and nutritional components.
