# Supplementary material for: Dynamic Changes in Egg Quality, Heritability and Correlation of These Traits and Yolk Nutrient throughout the Entire Laying Cycle
Source: Foods. 2023 Dec 14;12(24):4472. doi: 10.3390/foods12244472 (PMC10742422; doi:10.3390/foods12244472)
Supplement: Supplementary file 1 [file foods-12-04472-s001.zip › Supplementary S1.pdf]

Moisture Analysis:

(1) Take a clean weighing bottle, add 10 g of sea sands and a glass bar.
(2) Place in the 101°C - 105°C dryer to dry for about 1.0 h, take out, place into the dryer to cool for 0.5 h, and then weigh (m_3).
(3) Weigh 5 g ~ 10 g of yolks, place in the weighing bottle, use the small glass bar to well mix, and then weigh (m_1).
(4) Place in the 101°C - 105°C dryer to dry for 5 h, cover it and take it out, place in the dryer to cool for 0.5 h, then weigh (m_2). The moisture content in yolk can be calculated according to equation, where X is the moisture content in grams per 100 grams (g/100g).
The content of moisture in the yolk shall be calculated in accordance with formula:

$$X = (m_1-m_2)/(m_1-m_3)\times 100 \quad (1)$$

X: moisture content in sample, in grams per 100 grams (g/100g);
m_1: mass of weighing bottle (with sea sand, glass bar) and sample, in grams (g);
m_2: mass of weighing bottle (with sea sand, glass bar) and mass of dried sample, in grams (g);
m_3: mass of weighing bottle (with sea sand, glass bar), in grams (g);
100: the conversion coefficient.

Protein Analysis:

(1) Weigh a certain amount of a 2 g~5 g for yolk, place into a digestive tube, add 0.4 g of copper sulfate, 6 g of potassium sulphate and 20 mL of sulfuric acid into the digestion furnace for digestion.
(2) When the temperature of the digestion furnace reaches 420 ℃, continue digesting for 1 h, at this time the liquid in the digestive tube is in green and transparent state, take out and cool down, add 50 mL of water, and the automatic kjeldahl nitrogen analyzer can inject solution, distill, titrate, and record titration data automatically.
The content of protein in the yolk shall be calculated in accordance with formula:

$$X = \frac{(V_1 - V_2)\times c\times 0.0140}{m\times V_3/100}\times F\times 100 \quad (2)$$

X: the content of protein in the test sample, g/100 g;
V_1: the volume of the sulfuric acid or hydrochloric acid standard volumetric solution consumed by the test sample solution, mL;
V_2: the volume of the sulfuric acid or hydrochloric acid standard volumetric solution consumed by the reagent blank solution, mL;
C: the concentration of sulfuric acid or hydrochloric acid standard volumetric solution, mol/L;
0.0140: the mass of nitrogen equal to 1.0 mL of sulfuric acid [c((1/2)H2SO4) = 1.000 mol/L] or hydrochloric acid;
m: the mass of the test sample, g;
V_3: the volume of digestive solution pipetted, mL;
F: the coefficient of nitrogen conversion for protein, see Annex A for the nitrogen conversion coefficient of all kinds of foods;
100: the conversion coefficient.

Crude fat Analysis:

(1) Weigh 10g of yolk, place in a 50 mL test tube and add 10 mL of hydrochloric acid, put the test tube in the water bath at 70 ℃-80 ℃, and stir once very 5min–10min using a glass rod until the test sample is totally digested, about 40 min–50 min.

(2) Add 10 mL of ethanol in a test tube and mix. Cool, transfer the mixture in a 100mL measuring cylinder with stopper, wash the test tube with 25 mL of absolute ether for several times, and pour into the measuring cylinder together. Shake for 1 min with stopper after all the absolute ether is poured in the measuring cylinder, open the stopper, release gas, then plug it again, leave standing for 12 min, open the stopper carefully, and wash the fat adhered to the stopper and mouth of measuring cylinder with ether. Stand for 10 min-20 min, pipette supernatant liquid and place into the conical flask that has been dried to constant weight after top liquid is clear, add 5 mL of absolute ether into the measuring cylinder with stopper, shake, pipette the top ether after standing, and put in the original conical flask.

(3) Take the receiving flask down, recycle absolute ether or petroleum ether, evaporate to dry when there is 1 mL–2 mL solvent in the receiving flask, dry at 100 ℃±5 ℃ for 1 h, put in the dryer to cool for 0.5 h, and then weigh. Repeat the above operations until constant weight is reached.

The content of fat in the yolk shall be calculated in accordance with formula:

$$X=(m_1-m_0)/m_2\times100 \qquad (3)$$

X： the content of fat in the test sample, g/100g;

m_1： the mass of receiving flask and fat after constant weight, g;

m_0： the mass of receiving flask, g;

m_2： the mass of test sample, g;

100: the conversion coefficient.

Amino Acid Analysis:

(1) Weigh 3 grams of egg yolk sample and place it in a hydrolysis tube. Add 10mL to 15mL of 6 mol/L hydrochloric acid solution into the tube.

(2) Add 3 to 4 drops of phenol to the hydrolysis tube. Place the tube in a freezing agent, freeze it for 3 to 5 minutes, attach it to the suction tube of a vacuum pump, evacuate the tube (close to 0 Pa), then fill it with nitrogen gas. Repeat the vacuum–nitrogen-filling process three times, seal it while filled with nitrogen gas, and close the screw cap. Place the sealed hydrolysis tube in a constant temperature oven or hydrolysis furnace at 110°C±1°C and hydrolyze it for 22 hours. Remove it and allow for it to cool to room temperature.

(3) Open the hydrolysis tube and filter the hydrolysate into a 50mL volumetric flask. Wash the hydrolysis tube several times with a small amount of water, transferring the rinsing solution into the same 50mL volumetric flask. Finally, bring the volume up to the mark with water, and shake well to mix.

(4) Accurately transfer 1.0mL of the filtrate to a 15mL or 25mL test tube. Heat under reduced pressure at 40°C to 50°C using a test tube concentrator or parallel evaporator. After drying,

dissolve the residue in 1mL to 2mL of water, then subject it to vacuum drying and finally evaporate to dryness.

(5) Dissolve the dried residue in 1.0mL to 2.0mL of pH 2.2 sodium citrate buffer solution, shake well, and filter the solution through a 0.22μm membrane filter. Transfer the filtered solution to the instrument's sample vial for analysis.

(6) Inject the mixed amino acid standard working solution and sample test solution in equal volumes into the amino acid analyzer. Using an external standard method, determine the concentration of amino acids in the sample test solution based on peak areas.

The content of each amino acid in the mixed amino acid standard reserve solution shall be calculated in accordance with formula:

$$C_{\mathrm{j}} = \frac{\mathrm{m}_{\mathrm{j}}}{M_{\mathrm{j}} \times 250} \times 1000 \qquad (4)$$

j: the concentration of amino acid j in the mixed amino acid standard stock solution, measured in micromoles per milliliter (μmol/mL);

m_j: the mass of amino acid standard j taken, measured in milligrams (mg);

M_j: the molecular weight of amino acid standard j;

250: the volume made up to, measured in milliliters (mL);

1000: conversion factor.

The content of amino acids in the sample determination solution shall be calculated in accordance with formula:

$$C_{\mathrm{i}} = \frac{C_s}{A_s} \times A_i \qquad (5)$$

i: The content of amino acid i in the sample test solution, measured in nanomoles per milliliter (nmol/mL);

A_i: peak area of amino acid i in the sample test solution;

A_s: peak area of amino acid s in the amino acid standard working solution;

C_s: content of amino acid s in the amino acid standard working solution, measured in nanomoles per milliliter (nmol/mL).

The content of amino acid shall be calculated in accordance with formula:

$$Xi = \frac{ci \times F \times V \times M}{m \times 10^9} \times 100 \qquad (6)$$

i: content of amino acid i in the sample, measured in grams per 100 grams (g/100g);

ci: content of amino acid i in the sample test solution, measured in nanomoles per milliliter (nmol/mL);

F: dilution factor;
V: volume of transferred sample hydrolysate, measured in milliliters (mL);
M: molar mass of amino acid i, measured in grams per mole (g/mol). The names and molar masses of each amino acid are detailed in Table 2;
m: weight of sample, measured in grams (g);
109: conversion factor from nanograms (ng) to grams (g);
100: conversion factor.

Fatty Acid Analysis:

(1) Weigh 5g of egg yolk into a 250mL flat-bottomed flask, accurately add 2.0mL of undecanoic acid triglyceride internal standard solution. Add approximately 100mg of pyrogallol, a few pellets of boiling stones, then add 2mL of 95% ethanol and 4mL of water, mix well, and add 10mL of hydrochloric acid solution, mix thoroughly.

(2) Place the flask in a water bath at 70-80°C for 40 minutes for hydrolysis. Shake the flask every 10 minutes to ensure particles adhered to the flask walls mix into the solution. After hydrolysis, remove the flask and allow it to cool to room temperature.

(3) After hydrolysis, add 10mL of 95% ethanol to the sample, mix well. Transfer the hydrolyzed solution from the flask to a separating funnel, rinse the flask and stopper with a 50mL mixture of ether and petroleum ether, pour the rinsing solution into the separating funnel, cover, shake for 5 minutes, let it stand for 10 minutes. Collect the ether layer extract into a 250mL flask. Repeat the extraction of the hydrolyzed solution 3 times, finally rinse the separating funnel with a mixture of ether and petroleum ether and collect it in a 250mL flask. Concentrate using a rotary evaporator until dry, the residue is the fat extract.

(4) Add 8mL of 2% sodium hydroxide methanol solution to the fat extract, connect to a reflux condenser, reflux on a water bath at 80°C±1°C until oil droplets disappear. Add 7mL of 15% methanol boron trifluoride solution from the upper end of the reflux condenser, continue refluxing at 80°C±1°C for 2 minutes. Rinse the reflux condenser with a small amount of water. Stop heating, remove the flask from the water bath, quickly cool to room temperature. Accurately, add 10mL-30mL of n-heptane, shake for 2 minutes, then add saturated sodium chloride solution, allow to stand for layering. Pipette approximately 5mL of the upper n-heptane extract into a 25mL test tube, add about 3g-5g of anhydrous sodium sulfate, shake for 1 minute, let it stand for 5 minutes, and then pipette the upper layer solution into a sample vial for analysis.

(5) Inject individual fatty acid methyl ester standard solution and a mixed fatty acid methyl ester standard solution separately into the gas chromatograph for peak identification. Inject the fatty acid standard determination solution and sample determination solution into the gas chromatograph and quantitate by the peak area.

$$X_{\mathrm{i}} = F_i \times \frac{A_i}{A_{C11}} \times \frac{\rho_{\mathrm{C11}} \times \mathrm{V}_{\mathrm{C11}} \times 1.0067}{m} \times 100 \qquad (7)$$

X_i: content of fatty acid methyl ester i, expressed in grams per 100 grams (g/100g);
F_i: response factor of fatty acid methyl ester i;

A_i: peak area of fatty acid methyl ester i in the sample;
A_{C11}: peak area of the internal standard undecanoic acid methyl ester added to the sample;
ρ_{C11}: concentration of undecanoic acid triglyceride, in milligrams per milliliter (mg/mL);
V_{C11}: volume of undecanoic acid triglyceride added to the sample, in milliliters (mL);
1.0067: conversion factor of undecanoic acid triglyceride to undecanoic acid methyl ester;
m: mass of the sample, in milligrams (mg);
100: the conversion coefficient.

$$F_i = \frac{\rho_{Si} \times A_{11}}{A_{Si} \times \rho_{11}} \quad (8)$$

i: response factor of fatty acid methyl ester i;
ρ_{Si}: concentration of each fatty acid methyl ester i in the mixed standard solution, measured in milligrams per milliliter (mg/mL);
A_{11}: peak area of undecanoic acid methyl ester;
A_{Si}: peak area of fatty acid methyl ester i;
ρ_{11}: concentration of undecanoic acid methyl ester in the mixed standard solution, measured in milligrams per milliliter (mg/mL).

A single fatty acid methyl ester standard solution and a mixed fatty acid methyl ester standard solution were injected separately into the gas chromatograph for qualitative analysis of the chromatographic peaks.
a) Capillary column: Poly (cyanopropyl) siloxane strong polar stationary phase, column length 100m, inner diameter 0.25mm, film thickness 0.2μm.
b) Injector temperature: 270°C.
c) Detector temperature: 280°C.
d) Temperature program: Initial temperature 100°C, held for 13 minutes; 100°C to 180°C, ramp rate 10°C/min, held for 6 minutes; 180°C to 200°C, ramp rate 1°C/min, held for 20 minutes; 200°C to 230°C, ramp rate 4°C/min, held for 10.5 minutes.
e) Carrier gas: Nitrogen.
f) Split ratio: 100:1.
g) Injection volume: 1.0μL.
h) The detection conditions should meet a minimum theoretical plate number (n) of 2000/m and a resolution (R) of at least 1.25.
