# Supplementary figures and images for: Dynamic Changes in Egg Quality, Heritability and Correlation of These Traits and Yolk Nutrient throughout the Entire Laying Cycle
Source: Foods. 2023 Dec 14;12(24):4472. doi: 10.3390/foods12244472 (PMC10742422; doi:10.3390/foods12244472)

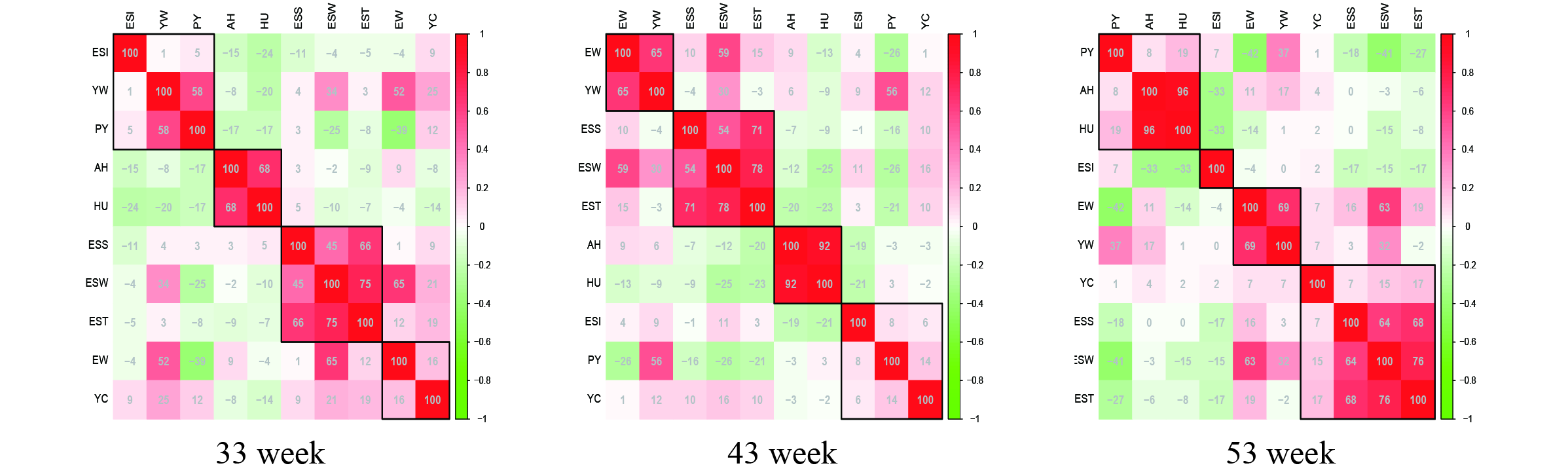

Supplement: Supplementary file 1 [file foods-12-04472-s001.zip › Figure S1. The correlation of the egg qualities at different weeks.tif]
